# Dual-specificity tyrosine phosphorylation-regulated kinase 1A ameliorates insulin resistance in neurons by up-regulating IRS-1 expression

**DOI:** 10.1074/jbc.RA119.010809

**Published:** 2019-11-13

**Authors:** Shijiao Tian, Wenming Jia, Mei Lu, Juan Zhao, Xiulian Sun

**Affiliations:** ‡ Department of Neurology, Qilu Hospital of Shandong University, No. 107 Wenhuaxi Rd., 250012 Jinan, China; § Brain Research Institute, Qilu Hospital of Shandong University, No. 107 Wenhuaxi Rd., 250012 Jinan, China; ¶ NHC Key Laboratory of Otorhinolaryngology, Chinese Ministry of Health, Qilu Hospital of Shandong University, No. 44 Wenhuaxi Rd., 250012 Jinan, China; ‖ Department of Geriatrics, Qilu Hospital of Shandong University, No. 107 Wenhuaxi Rd., 250012 Jinan, China; ** The Key Laboratory of Cardiovascular Remodeling and Function Research, Chinese Ministry of Education, Chinese National Health Commission, Qilu Hospital of Shandong University, No. 107 West Wenhua Rd., Jinan, 250012 Shandong Province, China

**Keywords:** insulin resistance, insulin receptor substrate 1 (IRS-1), DYRK1A, Alzheimer disease, diabetes, neurodegenerative disease, brain neuron, db/db mouse, metabolic disorder

## Abstract

Insulin resistance in the brain is a pathological mechanism that is shared between Alzheimer's disease (AD) and type 2 diabetes mellitus (T2DM). Although aberrant expression and phosphorylation of insulin receptor substrate 1 (IRS-1) contribute to insulin resistance, the underlying mechanism remains elusive. In this study, we used several approaches, including adeno-associated virus-based protein overexpression, immunoblotting, immunoprecipitation, immunohistochemistry, and *in situ* proximal ligation assays, to investigate the function of dual-specificity tyrosine phosphorylation-regulated kinase 1A (DYRK1A) in IRS-1 regulation and the downstream insulin signaling in neurons. We found that DYRK1A overexpression up-regulated IRS-1 expression by slowing turnover of the IRS-1 protein. We further observed that DYRK1A directly interacted with IRS-1 and phosphorylated IRS-1's multiple serine residues. Of note, DYRK1A and IRS-1 were coordinately up-regulated in the prefrontal cortex of *db*/*db* mice brain. Furthermore, DYRK1A overexpression ameliorated chronic high insulin-induced insulin resistance in SH-SY5Y cells as well as in primary rat neurons. These findings suggest that DYRK1A protects against insulin resistance in the brain by elevating IRS-1 expression.

## Introduction

Epidemiological and basic studies have revealed the close interactions between Alzheimer's disease (AD)^2^ and type 2 diabetes mellitus (T2DM). T2DM is considered to be a risk factor for AD (1). AD is proposed as “type 3 diabetes” for the extensive abnormalities of insulin signaling in the brain (2). However, the molecular mechanism underlying the interactions between these two prevalent diseases remains unclear. Brain insulin resistance is the common feature shared by AD and T2DM (3). T2DM animal models with cognitive deficits showed strong biochemical evidence of brain insulin resistance (4, 5). Substantial evidence also showed that brain insulin resistance encourages and even triggers key pathological events such as β-amyloid plaques and Tau phosphorylation in AD (6–8). Furthermore, intranasal insulin administration improved cognitive performance in both AD and T2DM patients with cognitive dysfunction (9–12).

Insulin binds to insulin receptor (IR) and facilitates the phosphorylation of insulin receptor substrate (IRS) in the brain (13). Upon tyrosine phosphorylation by insulin receptor, IRS will transduce insulin stimulation through divergent signaling pathways including the phosphoinositide 3-kinase/protein kinase B (AKT)/mTOR pathway. By contrast, specific serine phosphorylation of IRS by many protein kinases such as c-Jun NH_2_-terminal kinase (JNK) will inhibit insulin-induced tyrosine phosphorylation and thus disturb insulin signaling (14). Similarly, IRS degradation via a ubiquitin-proteasome pathway also promotes insulin resistance (15, 16). Insulin receptor substrate 1 (IRS-1) dysregulation has been observed in the brains of AD patients and AD mouse models (7). Abnormal serine phosphorylation or decreased expression of IRS-1 contributes to insulin resistance in the brain as well as in the peripheral tissues (3, 17). Drugs capable of ameliorating IRS-1 expression disorder exert neuroprotective effects through improving brain insulin signaling (18).

Dual-specificity tyrosine phosphorylation-regulated kinase 1A (DYRK1A) is a Ser/Thr protein kinase located in the Down syndrome critical region at chromosome 21. It is comprised of multiple domains, including a nuclear localization signal (NLS), a kinase domain, a proline-glutamic acid-serine-threonine–rich (PEST) domain, a consecutive histidine repeat (His) and a serine/threonine–rich domain (19). DYRK1A is abundantly expressed in the brain and interacts with numerous cytoskeletal, synaptic, and nuclear proteins in neurons (20). Through phosphorylating various substrates, DYRK1A participates in a broad spectrum of brain biological functions including the regulation of neural proliferation/differentiation, neurogenesis, dendritogenesis, and synaptogenesis (21, 22). Its aberrant expression contributes to the abnormal brain development in Down syndrome, and favors the pathological hallmarks of neurodegenerative diseases such as AD (23–27). Our recent study showed that DYRK1A is involved in neurodevelopment via RE1 silencing transcription factor and myocyte-specific enhance factor 2D (MEF2D) (28, 29). We also showed DYRK1A was degraded via a E3 ligase SCF^βTrCP^-mediated ubiquitin proteasome pathway (30). Moreover, we found DYRK1A phosphorylation of NFATc1 increased NFATc1 protein stability that is distinct with its regulation on NFATc2 (31). Pancreatic β-cells and neuronal cells share many similarities in terms of gene expression and development (32, 33). Inhibition of DYRK1A stimulates robust β-cell proliferation in adult primary islets and increases β-cell mass and improves glycemic control in mice (34–37). It would be interesting to further examine if DYRK1A regulates insulin resistance, particularly in the brain.

IRS-1 is the key molecule in insulin signaling and insulin resistance. Here our study demonstrated that DYRK1A physically interacts with IRS-1 and phosphorylates IRS-1. DYRK1A up-regulates IRS-1 protein expression by repressing its degradation. The protein levels of DYRK1A and IRS-1 are coordinately up-regulated in the prefrontal cortex (PFC) of *db*/*db* mice. Moreover, DYRK1A up-regulation of IRS-1 ameliorates insulin resistance induced by chronic high insulin exposure in SH-SY5Y cells and rat primary neurons. These data demonstrated DYRK1A as an important molecule in insulin resistance in the brain.

## Results

### DYRK1A increases IRS-1 protein expression

**Figure 1.**
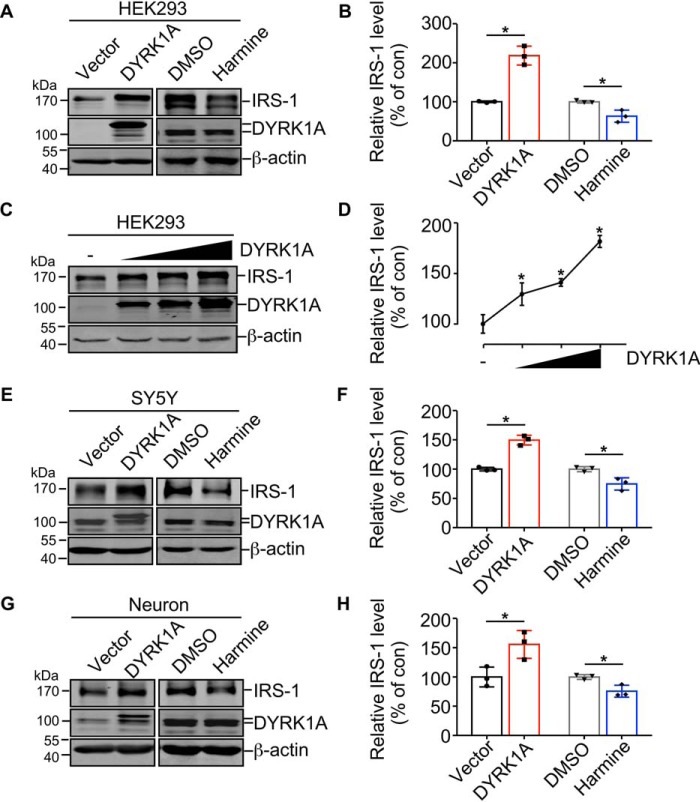
**DYRK1A up-regulates the IRS-1 protein level.** *A,* DYRK1A/harmine regulates the protein level of ectopic IRS-1 in HEK293 cells. HEK293 cells were co-transfected with pEnter-IRS-1 and pCMV6-entry or pCMV6-entry-DYRK1A (*left panels*). HEK293 cells were transfected with pEnter-IRS-1 and then treated with DMSO or 1 μm harmine for 24 h (*right panels*). IRS-1 and DYRK1A protein levels were examined by Western blot, β-actin was used as loading control. *B,* quantification of *A* using ImageJ software. The controls for DYRK1A or harmine were designated as 100%. Data are presented as mean ± S.D., *, *p* < 0.05, *p* values were calculated by Student's *t* test. *C,* DYRK1A increases the IRS-1 protein level in a dose-dependent manner. HEK293 cells were transfected with pEnter-IRS-1 and increasing amounts of pCMV6-entry-DYRK1A. Forty-eight hours after transfection, IRS-1 and DYRK1A protein levels were examined by Western blot, β-actin was used as loading control. *D,* quantification of *C*. Data are presented as mean ± S.D., *, *p* < 0.05, *p* values were calculated by one-way ANOVA followed by Tukey's multiple comparisons test (all DYRK1A-transfected groups compared with control group). *E,* DYRK1A/harmine regulates the protein level of endogenous IRS-1 in SH-SY5Y cells. SY5Y cells were transiently transfected with pCMV6-entry or pCMV6-entry-DYRK1A, or treated with DMSO or 1 μm harmine for 24 h. IRS-1 and DYRK1A protein levels were examined by Western blot, β-actin was used as loading control. *F,* quantification of *E*. The controls for DYRK1A or harmine were designated as 100%. Data are presented as mean ± S.D., *, *p* < 0.05, *p* values were calculated by Student's *t* test. *G,* DYRK1A/harmine regulates the protein level of endogenous IRS-1 in rat primary neurons. Rat primary neurons were infected with DYRK1A-coding AAV or control AAV, or treated with DMSO or 1 μm harmine for 24 h. IRS-1 and DYRK1A protein levels were examined by Western blot, β-actin was used as loading control. *H,* quantification of *G*. The controls for DYRK1A or harmine were designated as 100%. Data are presented as mean ± S.D., *, *p* < 0.05, *p* values were calculated by Student's *t* test. All quantified results were obtained from three independent experiments.

IRS-1 is a key molecule in insulin signaling and its down-regulation leads to insulin resistance. To investigate if DYRK1A affects insulin signaling, IRS-1 expression was examined in HEK293 cells overexpressing DYRK1A. Results showed that ectopic DYRK1A expression markedly increased the IRS-1 protein level to 218.5 ± 14.0% of control (Fig. 1, *A* and *B*, *lane 2 versus 1*; *p* = 0.0011). DYRK1A inhibitor harmine (38) repressed IRS-1 expression to 63.2 ± 9.0% of control in HEK293 cells (Fig. 1, *A* and *B*, *lane 4 versus 3*; *p* = 0.0159). We also observed that the IRS-1 protein level was increased in a dose-dependent manner with an increased DYRK1A expression level in HEK293 cells (Fig. 1, *C* and *D*). Endogenous IRS-1 expression was increased by DYRK1A to 149.6 ± 4.8% of control (Fig. 1, *E* and *F*, *lane 2 versus 1*; *p* = 0.0006) and decreased by DYRK1A inhibitor harmine to 74.8 ± 6.1% of control (Fig. 1, *E* and *F*, *lane 4 versus 3*; *p* = 0.0181) in neuroblastoma SH-SY5Y cells. Similar results were obtained in E18 primary rat neurons. DYRK1A expression up-regulated the IRS-1 protein level to 155.5 ± 13.8% of control (Fig. 1, *G–H*, *lane 2 versus 1*; *p* = 0.0300) and harmine down-regulated the IRS-1 protein level to 75.7 ± 5.9% of control (Fig. 1, *G* and *H*, *lane 4 versus 3*; *p* = 0.0183) in primary rat neurons. These results demonstrated that DYRK1A regulates IRS-1 protein expression.

### DYRK1A stabilizes IRS-1 by decreasing IRS-1 ubiquitination

**Figure 2.**
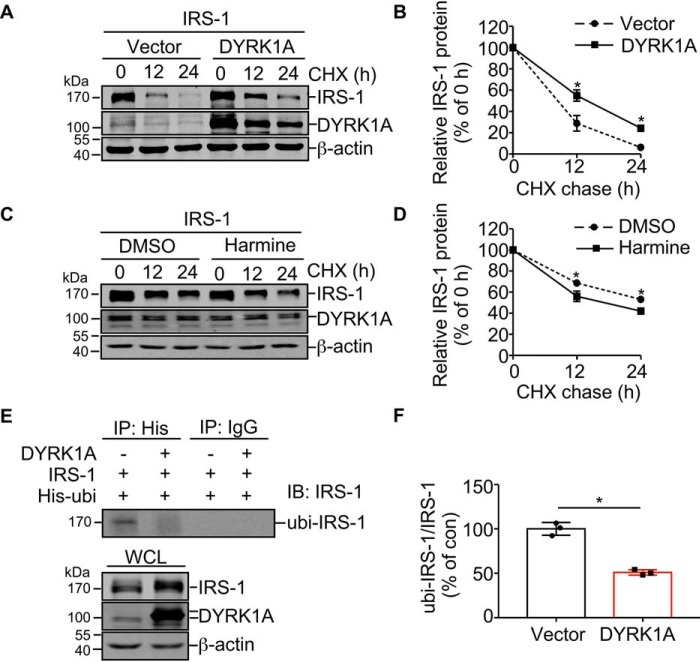
**DYRK1A stabilizes IRS-1 protein turnover.** *A,* DYRK1A influences the degradation rate of IRS-1 protein. HEK293 cells were co-transfected with pEnter-IRS-1 and pCMV6-entry or pCMV6-entry-DYRK1A. Twenty-four hours after transfection, cells were treated with 300 μg/ml of CHX for different times as indicated. Cell lysates were detected for IRS-1 and DYRK1A by Western blot, β-actin was used as loading control. *B,* quantification of *A*. All quantified results were obtained from three independent experiments. Data are presented as mean ± S.D., *, *p* < 0.05, *p* values between two groups were calculated by Student's *t* test at the same time point. *C,* harmine influences the degradation rate of IRS-1 protein. HEK293 cells were transfected with pEnter-IRS-1, and then treated with 300 μg/ml of CHX for different times as indicated. All cells were treated with DMSO or 1 μm harmine for 24 h before harvest. Cell lysates were detected for IRS-1 and DYRK1A by Western blot, β-actin was used as loading control. *D,* quantification of *C*. All quantified results were obtained from three independent experiments. Data are presented as mean ± S.D., *, *p* < 0.05, *p* values between two groups were calculated by Student's *t* test at the same time point. *E,* DYRK1A influences ubiquitination of IRS-1. HEK293 cells were co-transfected with pHis-ubi and IRS-1, with or without DYRK1A. Cell lysates were precipitated by anti-His or anti-mouse IgG antibody (negative control). The precipitations were detected for IRS-1 and whole cell lysates (*WCL*) were detected for IRS-1 and DYKR1A by Western blot. *F,* quantification of *E*. Quantified results were obtained from three independent experiments. Data are presented as mean ± S.D., *, *p* < 0.05, *p* values were calculated by Student's *t* test.

To examine if DYRK1A increases the IRS-1 protein by regulating IRS-1 protein turnover, HEK293 cells were co-transfected with IRS-1-FlagHis in the presence or absence of DYRK1A-MycFlag and then chased with cycloheximide. Results revealed that overexpression of DYRK1A stabilized IRS-1 protein turnover (Fig. 2, *A* and *B*). Reversely, DYRK1A inhibition by harmine destabilized IRS-1 proteins (Fig. 2, *C* and *D*). Previous reports showed that IRS-1 was degraded by a ubiquitin-proteasome pathway (39–41). To further elucidate the mechanism of IRS-1 stabilization by DYRK1A, IRS-1 ubiquitination was examined in HEK293 cells. Although the IRS-1 level was increased in whole cell lysates from cells transfected with DYRK1A, the ratio of ubiquitinated IRS-1 to total IRS-1 was significantly decreased by DYRK1A to 50.9 ± 1.8% of control (Fig. 2, *E* and *F*, *lane 2 versus 1*; *p* = 0.0004). Taken together, these results demonstrated that DYRK1A stabilized IRS-1 through decreasing IRS-1 ubiquitination and subsequent protein degradation.

To exclude whether DYRK1A up-regulates IRS-1 protein through gene transcription, IRS-1 mRNA levels were examined in HEK293, SH-SY5Y cells, and primary rat neurons. Neither overexpression nor inhibition of DYRK1A affected IRS-1 mRNA levels as analyzed by quantitative real-time PCR (data not shown), which indicated that DYRK1A regulates IRS-1 only at the protein level.

### DYRK1A interacts with IRS-1 and phosphorylates IRS-1 at Ser-312 and Ser-616

**Figure 3.**
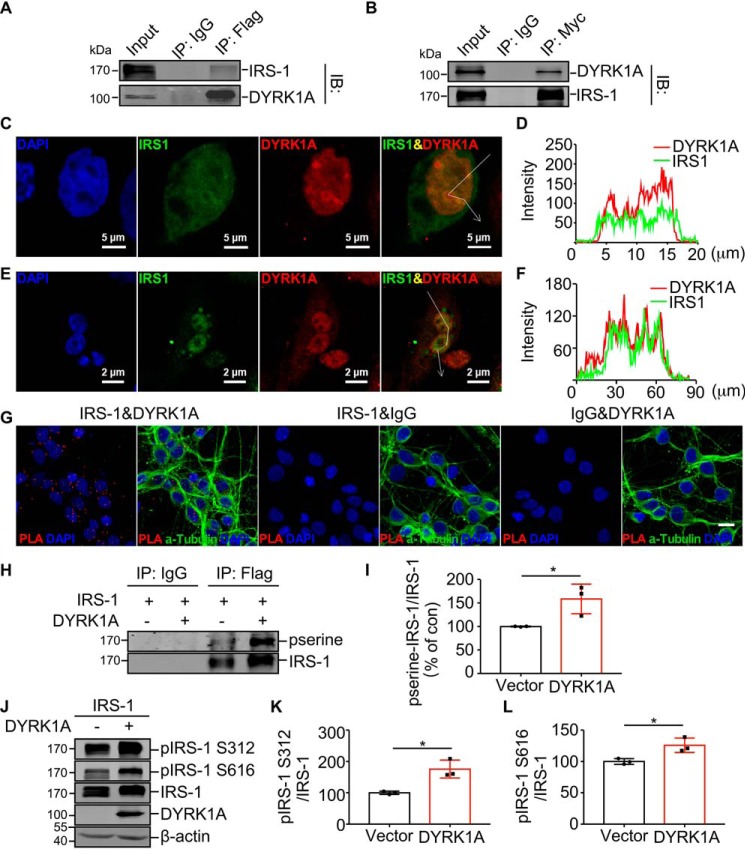
**DYRK1A interacts with IRS-1 and phosphorylates IRS-1 in cells.** *A* and *B,* IRS-1 interacts with DYRK1A. Cell lysates from HEK293 cells transfected with IRS-1-Myc and DYRK1A-FlagHis were immunoprecipitated with anti-FLAG (M2) affinity gel (*A*) or anti-c-myc affinity gel. *B,* the precipitates were detected for DYRK1A and IRS-1 protein by Western blot. *C,* DYRK1A co-localizes with IRS-1 in HEK293 cells. HEK293 cells transfected with IRS-1-Myc and DYRK1A-FlagHis were stained with anti-DYRK1A (Alexa Fluor 594, *red*) and anti-IRS-1 (Alexa Fluor 488, *green*) antibodies, DAPI (*blue*) was used to indicate the nucleus. Images were captured by a LSM 780 fluorescent confocal microscope. The *white line with an arrow* in the fourth picture indicates the selected line used in co-localization analysis. *D,* co-localization analysis of *C. E,* DYRK1A co-localizes with IRS-1 in T98G cells. Endogenous proteins were stained with anti-DYRK1A (Alexa Fluor 594, *red*) and anti-IRS-1 (Alexa Fluor 488, *green*) antibodies, DAPI (*blue*) was used to indicate the nucleus. Images were captured by a LSM 780 fluorescent confocal microscope. The *white line with an arrow* in the fourth picture indicates the selected line used in co-localization analysis. *F,* co-localization analysis of *E. G,* representative confocal images of a PLA between IRS-1 and DYRK1A (*red puncta*) in rat primary neurons. Alexa Fluor® 488 conjugate α-tubulin (*green*) was used to show the cytoskeleton and DAPI (*blue*) to the nucleus, *scale bar* 10 μm. Images were captured by a LSM 780 fluorescent confocal microscope. Maximum intensity projections of Z-stacks were performed to observe the maximum amount of PLA puncta. Negative PLA control was performed with IRS-1 or DYRK1A antibody with IgG. *H,* DYRK1A influences IRS-1 serine phosphorylation. Cell lysates from HEK293 cells overexpressing IRS-1-FlagHis with or without DYRK1A were immunoprecipitated with anti-FLAG (M2) affinity gel. Serine-phosphorylated IRS-1 and total IRS-1 were analyzed by Western blot. *I,* quantification of *H. J,* DYRK1A influences IRS-1 Ser-312 and IRS-1 Ser-616 phosphorylation. HEK293 cells were co-transfected with pEnter-IRS-1 and pCMV6-entry or pCMV6-entry-DYRK1A. Protein levels of pIRS-1 Ser-312, pIRS-1 Ser-616, IRS-1, and DYRK1A were detected by Western blot, β-actin was used as loading control. *K–L*, quantification of *I*. All quantified results were obtained from three independent experiments. Data are presented as mean ± S.D., *, *p* < 0.05, *p* values were calculated by Student's *t* test.

To further investigate the molecular mechanism of DYRK1A regulation of IRS-1 expression, the protein-protein interaction was examined with co-IP and co-localization. IRS-1-Myc and DYRK1A-FlagHis were co-expressed in HEK293 cells. Co-IP assay showed that anti-FLAG (M2) affinity gel efficiently precipitated IRS-1 (Fig. 3*A, lane 3*). Reciprocally, anti-c-myc affinity gel precipitated the DYRK1A protein, indicating the physical interaction of DYRK1A and IRS-1 in cells (Fig. 3*B, lane 3*).

To further validate this interaction, a immunofluorescent assay was conducted. Previous studies demonstrated that both DYRK1A and IRS-1 are expressed in cytoplasm and nucleus and their localization propensity varies in different cell types (38–40). HEK293 cells were transiently transfected with plasmids encoding IRS-1 and DYRK1A. We observed that ectopic DYRK1A was mainly localized in the nucleus. Although IRS-1 is expressed predominantly in cytoplasm, its expression was observed in both cytoplasm and nucleus in the minority of cells. In those HEK293 cells that have nuclear IRS-1, DYRK1A co-localized with IRS-1 in the nucleus (Fig. 3, *C* and *D*). Furthermore, endogenous DYRK1A and IRS-1 were detected in T98G cells. Different from their expression pattern in HEK293 cells, both DYRK1A and IRS-1 were predominantly expressed in the nucleus in T98G cells. Co-localization analysis showed that DYRK1A and IRS-1 were co-localized in the nucleus in T98G cells (Fig. 3, *E* and *F*). *In situ* proximal ligation assay (PLA) was also conducted to examine the co-localization of DYRK1A and IRS-1 in rat primary neurons. Consistent with previous studies (42), DYRK1A was observed in both cytoplasm and nucleus in neurons. PLA puncta of these two proteins were observed in both cytoplasm and nucleus in neurons (Fig. 3*G*). These results confirmed that DYRK1A physically interacts with IRS-1 in cells.

DYRK1A is a Ser/Thr protein kinase that phosphorylates APP, PS1, Tau, and many other key molecules involved in neuronal functions. Based on the fact that DYRK1A and IRS-1 interact with each other and IRS-1 is a protein with numerous Ser/Thr (43), we assumed that DYRK1A could phosphorylate IRS-1. To verify this hypothesis, HEK293 cells were transiently transfected with IRS-1 expression plasmid with a FLAG tag, either in the presence or absence of DYRK1A. Next, IRS-1 was precipitated by anti-FLAG M2 affinity gel, and anti-phosphoserine antibody was used to detect serine-phosphorylated IRS-1. This result showed that IRS-1 serine phosphorylation was elevated by DYRK1A to 158.4 ± 18.1% of control (Fig. 3, *H* and *I*; *p* = 0.0323), indicating that DYRK1A could phosphorylate the serine sites of IRS-1. Among numerous serine sites in IRS-1, Ser-312 and Ser-616 are two of the most studied residues regarding to protein degradation (44). To determine whether DYRK1A phosphorylates IRS-1 on Ser-312 and Ser-616, HEK293 cells were transfected with IRS-1 in the presence or absence of DYRK1A, pIRS-1 Ser-312, and pIRS-1 Ser-616 were detected with phosphor-residue–specific antibody by Western blotting. DYRK1A increased the pIRS-1 Ser-312 level to 175.6 ± 16.5% of control (Fig. 3, *J* and *K*, *lane 2 versus 1*; *p* = 0.0108). DYRK1A also increased the pIRS-1 Ser-616 level to 125.8 ± 6.6% of control (Fig. 3, *J* and *L*, *lane 2 versus 1*; *p* = 0.0224).

### DYRK1A and IRS-1 are coordinately up-regulated in PFC of db/db mice at 12 weeks of age

**Figure 4.**
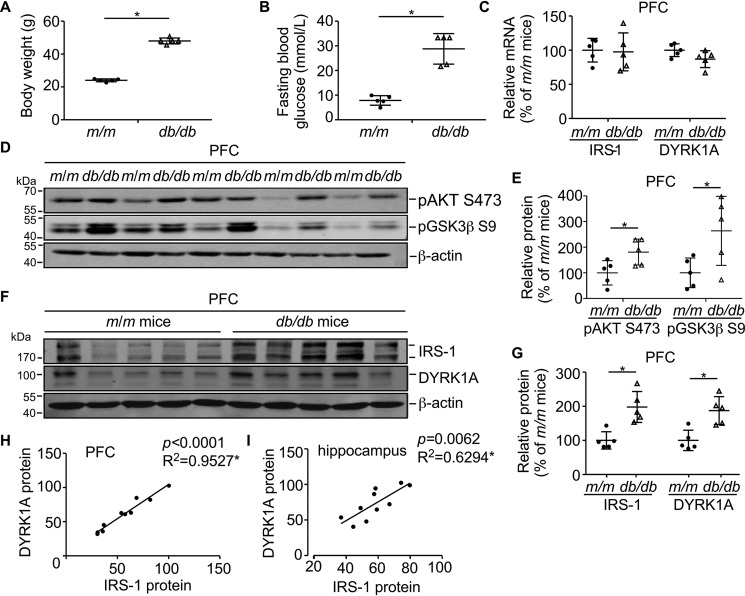
**DYRK1A and IRS-1 are coordinately expressed in *db*/*db* mice brain.** *A,* body weights of 12-week-old *db*/*db* mice are significantly higher than that of *m*/*m* mice. *B,* blood glucose (fasted for 6 h) of 12-week-old *db*/*db* mice are significantly higher than control *m*/*m* mice. For the glucose level exceeding the measure range of the blood glucose meter, the maximum measurement value of the meter (33.3 mmol/liter) was recorded. *C,* IRS-1 and DYRK1A mRNA expression do not change in *db*/*db* mice brain. PFC of 12-week-old *m*/*m* and *db*/*db* mice were detected for IRS-1 and DYRK1A mRNA. Data are presented as mean ± S.D., *n* = 5, *, *p* < 0.05, *p* values were calculated by Student's *t* test. *D,* pAKT Ser-473 and pGSK3β Ser-9 protein expression in mice brain. PFC of 12-week-old *m*/*m* and *db*/*db* mice were detected for pAKT Ser-473 and pGSK3β Ser-9 proteins, β-actin was used as loading control. *E,* quantification of *A*. The protein expression of pAKT Ser-473 and pGSK3β Ser-9 were designated as 100% in *m*/*m* mice. Data are presented as mean ± S.D., *n* = 5, *, *p* < 0.05, *p* values were calculated by Student's *t* test. *F,* IRS-1 and DYRK1A protein expression in mice brain. PFC of 12-week-old *m*/*m* and *db*/*db* mice were detected for IRS-1 and DYRK1A proteins, β-actin was used as loading control. *G,* quantification of *C*. The protein expression of IRS-1 and DYRK1A were designated as 100% in *m*/*m* mice. Data are presented as mean ± S.D., *n* = 5, *, *p* < 0.05, *p* values were calculated by Student's *t* test. *H,* DYRK1A and IRS-1 protein are coordinately expressed in PFC of mice brain. *n* = 10, *, *p* < 0.0001, *R*^2^ = 0.9527, *p* value was determined by Pearson's correlation test. *I,* DYRK1A and IRS-1 protein are coordinately expressed in the hippocampus of mice brain. *n* = 10, *, *p* = 0.0062, *R*^2^ = 0.6294, *p* value was determined by Pearson's correlation test.

To further examine the association between DYRK1A and IRS-1 *in vivo*, we detected their mRNA and protein expression at 12 weeks of age in *m*/*m* and *db*/*db* mouse brains. Consistent with previous reports (47), *db*/*db* mice were already obese and hyperglycemic compared with 12-week-old *m*/*m* mice (Fig. 4, *A* and *B*). PFC and hippocampus were dissected from mice brains and then investigated for DYRK1A and IRS-1 expression. DYRK1A and IRS-1 mRNA levels did not differ significantly in PFC between *m*/*m* and *db*/*db* mice (Fig. 4*C*, *p* = 0.0.2690 for DYRK1A and *p* = 0.8365 for IRS-1). We observed that pAKT Ser-473 and pGSK3β Ser-9 were elevated in *db*/*db* mice compared with *m*/*m* mice in PFC at 12 weeks of age, indicating the existence of disturbed insulin signaling (*p* = 0.0005 and *p* = 0.0072, respectively) (Fig. 4, *D* and *E*). Interestingly, DYRK1A protein was increased to 187.5 ± 22.7% (*p* = 0.0016) and IRS-1 protein was increased to 197.9 ± 23.3% (*p* = 0.0006) in PFC of *db*/*db* mice compared with *m*/*m* mice at 12 weeks of age (Fig. 4, *F* and *G*). We found significant positive correlations between DYRK1A and IRS-1 protein levels in both PFC (Fig. 4*H*, *p* < 0.0001, *R*^2^ = 0.9527 by Pearson's correlation test) and hippocampus (Fig. 4*I*, *p* = 0.0062, *R*^2^ = 0.6294 by Pearson's correlation test) at 12 weeks of age, suggesting that the two proteins might function coordinately in these brain areas in insulin signaling.

### DYRK1A is increased in insulin-resistant cell models

**Figure 5.**
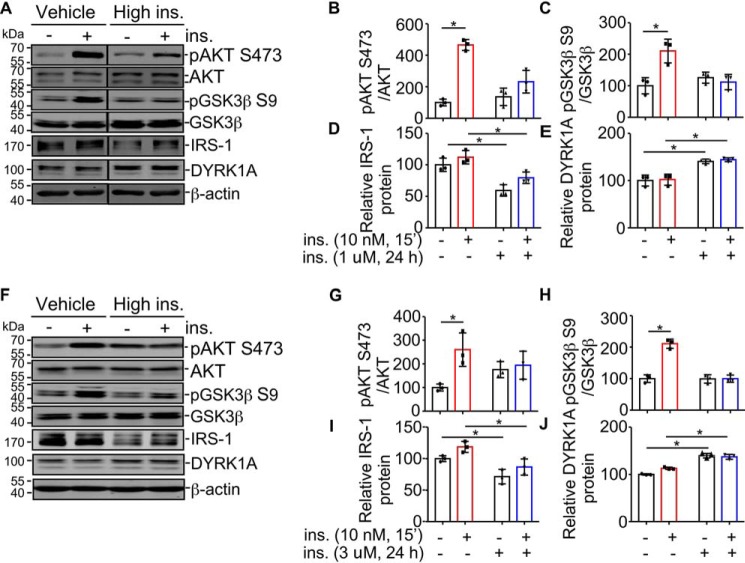
**DYRK1A is increased in insulin-resistant cell models.** *A,* establishment of the insulin-resistant model in SH-SY5Y cells. *Vehicle* indicates absence of insulin and *High ins* indicates a chronic high insulin condition (1 μm for 24 h). After pretreatment, the media were removed and the cells were washed three times and then rechallenged with 10 nm insulin for 15 min. Levels of pAKT Ser-473, AKT, pGSK3β Ser-9, GSK3β, IRS-1, and DYRK1A were detected by Western blot, β-actin was used as loading control. *B–E,* quantification of *A. F,* establishment of the insulin-resistant model in primary neurons. *Vehicle* indicates the absence of insulin and *High ins* indicates a chronic high insulin condition (3 μm for 24 h). After pretreatment, the media were removed and the cells were washed three times and then rechallenged with 10 nm insulin for 15 min. Levels of pAKT Ser-473, AKT, pGSK3β Ser-9, GSK3β, IRS-1, and DYRK1A were detected by Western blot, β-actin was used as loading control. *G–J,* quantification of *F*. All quantified results were obtained from three independent experiments. Data are presented as mean ± S.D., *, *p* < 0.05, *p* values were calculated by Student's *t* test (for pAKT Ser-473/AKT and pGSK3β Ser-9/GSK3β changes after 10 nm insulin stimulation) or two-way ANOVA followed by Tukey's multiple comparisons test (for IRS-1 and DYRK1A changes at different conditions).

IRS-1 is a key molecule transducing insulin signaling and its decreased expression is often observed in the context of IR (17, 45). Because DYRK1A is closely correlated with IRS-1 and is capable of up-regulating IRS-1 expression, we next explored the impact of DYRK1A on insulin signaling, especially in the insulin-resistant state. Defined as attenuated responsivity to insulin stimulation, IR could be induced by chronic high insulin treatment (16). SH-SY5Y cells were cultured in serum-free DMEM without or with 1 μm insulin for 24 h to achieve basal or IR states, respectively. Then cells were shortly stimulated with 10 nm insulin for another 15 min. pAKT Ser-473/AKT and pGSK3β Ser-9/GSK3β ratio changes induced by acute insulin stimulation were used to reflect insulin responsiveness. At basal state, acute insulin stimulation triggered elevation of pAKT Ser-473/AKT by 365.7 ± 23.8% (Fig. 5*A*, *panels 1* and *2,* and *B, lane 2 versus 1*; *p* = 0.0001) and pGSK3β Ser-9/GSK3β by 110.3 ± 26.4% of controls (Fig. 5, *A*, *panels 3* and *4,* and *C, lane 2 versus 1*; *p* = 0.0140), respectively, indicating high insulin responsiveness. In contrast, after chronic insulin pretreatment, the acute insulin stimulation-induced pAKT Ser-473/AKT increase was reduced to a level of no statistical significance (Fig. 5, *A, panels 1* and *2*, *B*, *lane 4 versus 3*; *p* = 0.1412) and no noticeable change was observed from pGSK3β Ser-9/GSK3β (Fig. 5, *A*, *panels 3* and *4,* and *C, lane 4 versus 3*; *p* = 0.4650), suggesting the successful induction of insulin resistance. Consistent with previous findings (15, 46), we observed chronic insulin treatment decreased and acute insulin stimulation increased IRS-1 protein (Fig. 5, *panel 5*, and *D*). Of interest, we found that DYRK1A proteins were elevated by chronic insulin treatment (∼140% of basal with or without acute insulin stimulation, Fig. 5, *A, panel 6*, and *E*, *p* = 0.0016 and *p* = 0.0022, respectively). DYRK1A mRNA levels were also examined following chronic insulin exposure, but no noticeable difference in gene transcription was detected (data not shown), excluding the possibility that DYRK1A changes were from the transcriptional level.

Insulin-resistant rat primary neuron cultures were also established. Our results demonstrated that acute insulin-stimulation induced pAKT Ser-473/AKT and pGSK3β Ser-9/GSK3β elevations were efficiently sabotaged by exposing to 3 μm insulin for 24 h in rat primary neurons (Fig. 5, *F–H*). As expected, IRS-1 expression was down-regulated after chronic high insulin treatment (Fig. 5, *F, panel 5*, and *I*). Conversely, DYRK1A was up-regulated by chronic insulin treatment (to ∼139.7% of basal state without acute insulin stimulation, *p* = 0.0005; and ∼124.2% of basal state with acute insulin stimulation, *p* = 0.0119; Fig. 5, *F, panel 6*, and *J*). No noticeable difference was found in DYRK1A mRNA levels (data not shown). These results demonstrated that DYRK1A protein levels were increased by insulin resistance *in vitro*. However, the underlying mechanism and the consequences of its up-regulation in insulin resistance are still not clear.

### DYRK1A ameliorates chronic high insulin-induced insulin resistance in SH-SY5Y cells

**Figure 6.**
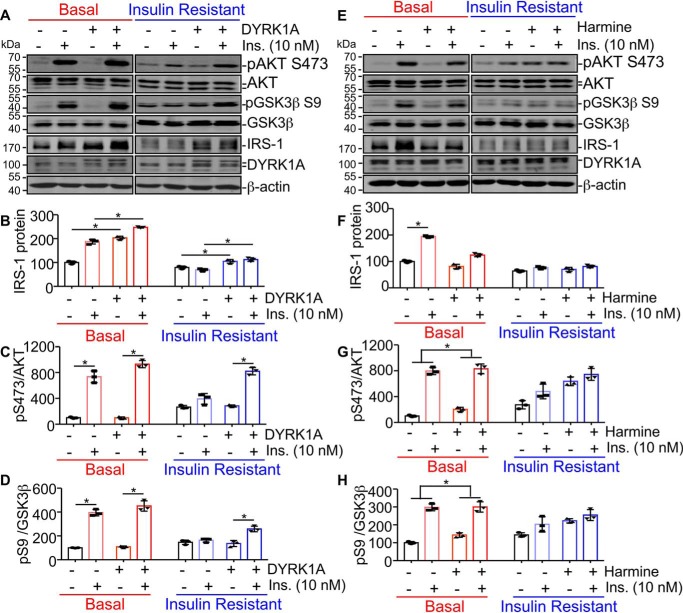
**DYRK1A ameliorates chronic high insulin-induced insulin resistance in SH-SY5Y cells.** *A,* SH-SY5Y cells were transfected with DYRK1A or control plasmid and then treated in vehicle or high ins. conditions for 24 h to induce insulin resistance, and then rechallenged with 10 nm insulin for 15 min. Levels of pAKT Ser-473, AKT, pGSK3β Ser-9, GSK3β, IRS-1, DYRK1A, and β-actin were detected by Western blot. *B–D*, quantification of *A*. All quantified results were obtained from three independent experiments. Data are presented as mean ± S.D., *, *p* < 0.05, *p* values were calculated by Student's *t* test (for *C* and *D*) or two-way ANOVA followed by Tukey's multiple comparisons test (for *B*). *E,* SH-SY5Y cells were treated with DMSO or 1 μm harmine in vehicle or high ins. conditions for 24 h, and then rechallenged with 10 nm insulin for 15 min. Levels of pAKT Ser-473, AKT, pGSK3β Ser-9, GSK3β, IRS-1, DYRK1A, and β-actin were detected by Western blot. *F–H*, the quantification of *C*. All quantified results were obtained from three independent experiments. Data are presented as mean ± S.D., *, *p* < 0.05, *p* values were calculated by Student's *t* test (for *G* and *H*) or two-way ANOVA followed by Tukey's multiple comparisons test (for *F*).

Based on the reliable IR models, we next explored the impact of DYRK1A on insulin signaling at both basal and IR states. SH-SY5Y was transfected with DYRK1A expressing plasmid or its vector control. IRS-1 expression and insulin responsiveness were measured under both basal and IR states. We found DYRK1A up-regulated IRS-1 expression both in basal and IR states (Fig. 6, *A* and *B, lane 3 versus 1*, *lane 4 versus 2*, *lane 7 versus 5*, and *lane 8 versus 6*). DYRK1A up-regulated IRS-1 expression to 203.2 ± 5.6% of control in the basal state (Fig. 6, *A* and *B*, *lane 3 versus 1*, *p* < 0.0001), and to 132.5 ± 5.6% of control in IR states (Fig. 6, *A* and *B*, *lane 7 versus 5, p* = 0.0056). Furthermore, both control and DYRK1A-transfected cells displayed high insulin responsiveness with remarkable pAKT Ser-473/AKT elevations at the basal state (Fig. 6, *A* and *C*, *lane 2 versus 1* and *lane 4 versus 3*). The high insulin responsiveness was confirmed with increased pGSK3β Ser-9/GSK3β after insulin stimulation (Fig. 6, *A* and *D*, *lane 2 versus 1* and *lane 4 versus 3*). When cells were previously treated with 1 μm insulin for 24 h before stimulation with 10 nm insulin for 15 min, Ser-473 AKT or Ser-9 GSK3β phosphorylation were unchanged after insulin stimulation, indicating the successful induction of insulin-resistant states (Fig. 6, *A, C,* and *D*, *lane 6 versus 5*). However, in cells transfected with DYRK1A, moderate insulin responsiveness was observed with increased levels of Ser-473 AKT phosphorylation (288.5 ± 4.0% of control, *p* < 0.0001), indicating DYRK1A ameliorate insulin resistance (Fig. 6, *A* and *C*, *lane 8 versus 7*). Similar results were confirmed with increased Ser-9 GSK3β phosphorylation in cells expressing DYRK1A (189.1 ± 9.7% of control, *p* = 0.0036, Fig. 6, *A* and *D, lane 8 versus 7*). These data implied that DYRK1A can ameliorate insulin resistance possibly through up-regulation of IRS-1.

To further validate the effect of DYRK1A on insulin signaling, harmine was added to SH-SY5Y cells to inhibit DYRK1A. Reduction of IRS-1 expression was observed in the harmine-treated group at basal state (Fig. 6, *E* and *F, lane 3 versus 1*, *lane 4 versus 2*, *p* = 0.0154 and *p* < 0.0001, respectively), whereas no noticeable change was shown at the IR state (Fig. 6, *E* and *F, lane 7 versus 5*, *lane 8 versus 6*). Consistent with previous reports (46), 10 nm insulin stimulation for 15 min increased IRS-1 expression (Fig. 6, *E* and *F, lane 2 versus 1*). Harmine treatment diminished the IRS-1 increase induced by insulin (Fig. 6, *E* and *F, lanes 1–4*, *p* < 0.0001). IRS-1 expression was greatly reduced in the insulin-resistant state and the increase after acute insulin stimulation was also abolished with or without harmine (Fig. 6, *E* and *F, lane 5-8*). The increase of pAKT Ser-473/AKT and pGSK3β Ser-9/GSK3β after 10 nm insulin stimulation indicated the responsiveness to insulin at the basal state (Fig. 6, *E*, *G,* and *H*, *lane 2 versus 1*, *lane 4 versus 3*). Of note, harmine remarkably reduced the ratio changes of pAKT Ser-473/AKT and pGSK3β Ser-9/GSK3β in the basal state (change between Fig. 6, *E*, *G,* and *H*, *lanes 4* and *3 versus* change between *lanes 2* and *1, p* = 0.0039 for pAKT Ser-473/AKT ratio changes and *p* < 0.0001 for pGSK3β Ser-9/GSK3β ratio changes, respectively), indicating harmine attenuated insulin responsiveness at the basal state. At the insulin-resistant state, no increase could be observed in AKT phosphorylation or GSK3β phosphorylation after insulin stimulation, indicating the successful induction of insulin resistance (Fig. 6, *E*, *G,* and *H*, *lane 6 versus 5*). Harmine could not affect the insulin responsiveness in the insulin-resistant state (Fig. 6, *E*, *G,* and *H, lanes 5-8*). These data suggested that harmine treatment diminished insulin responsiveness at the basal state, the possible underlying mechanism might be its decrease of IRS-1 proteins.

### DYRK1A ameliorates chronic high insulin-induced insulin resistance in primary rat neurons

**Figure 7.**
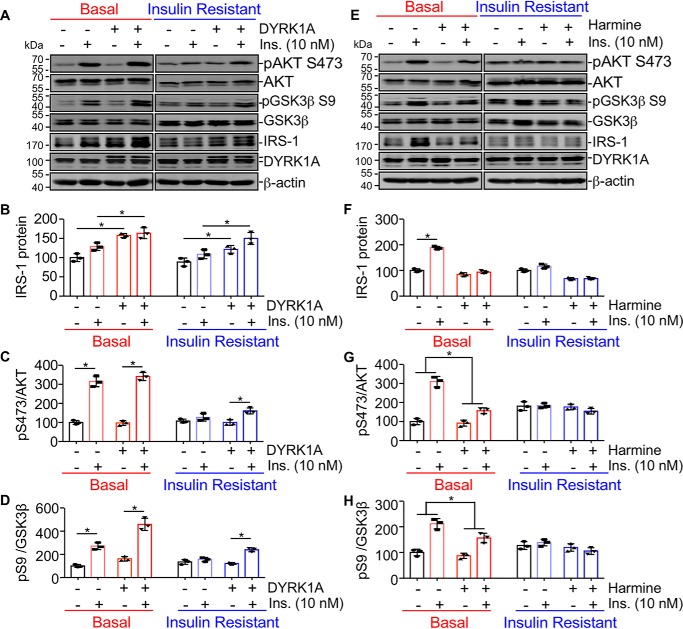
**DYRK1A ameliorates chronic high insulin-induced insulin resistance in primary neurons.** *A,* primary E18 neurons were infected with DYRK1A encoding or control AAV and then treated in vehicle or high ins. conditions (3 μm) for 24 h, and then rechallenged with 10 nm insulin for 15 min. Levels of pAKT Ser-473, AKT, pGSK3β Ser-9, GSK3β, IR-1, DYRK1A, and β-actin were detected by Western blot. *B–D,* the quantification of *A*. All quantified results were obtained from three independent experiments. Data are presented as mean ± S.D., *, *p* < 0.05, *p* values were calculated by Student's *t* test (for *C* and *D*) or two-way ANOVA followed by Tukey's multiple comparisons test (for *B*). *E,* primary neurons were treated with DMSO or 1 μm harmine in vehicle or high ins. conditions (3 μm) for 24 h, and then rechallenged with 10 nm insulin for 15 min. Levels of pAKT Ser-473, AKT, pGSK3β Ser-9, GSK3β, IRS-1, DYRK1A- and β-actin were detected by Western blot. *F–H,* quantification of *C*. All quantified results were obtained from three independent experiments. Data are presented as mean ± S.D., *, *p* < 0.05, *p* values were calculated by Student's *t* test (for *G* and *H*) or two-way ANOVA followed by Tukey's multiple comparisons test (for *F*).

To further determine the role of DYRK1A in insulin signaling, primary neurons were infected with rAAV-2/9 expressing DYRK1A. Three days after infection, IRS-1 expression and insulin sensitivity were tested under both basal and IR states. DYRK1A elevated IRS-1 expression to 157.5 ± 8.4% of control at basal states (Fig. 7, *A* and *B, lane 3 versus 1*, *p* = 0.0006), and to 137.4 ± 9.9% of control at IR states (Fig. 7, *A* and *B, lane 7 versus 5*, *p* = 0.0145). Similar elevations of IRS-1 by DYRK1A were achieved in neurons stimulated with 10 nm insulin in both basal and IR states (Fig. 7, *A* and *B, lane 4 versus 2*, *lane 8 versus 6*). Consistent with our results in SH-SY5Y cells, acute insulin stimulation markedly increased pAKT Ser-473/AKT and pGSK3β Ser-9/GSK3β at the basal state (Fig. 7, *A*, *C,* and *D*), indicating the neurons respond to insulin. Similarly, chronic insulin exposure (3 μm insulin for 24 h) eliminated the elevations of pAKT Ser-473/AKT and pGSK3β Ser-9/GSK3β induced by acute insulin treatment (10 nm, 15 min) in the control group (Fig. 7, *A, C,* and *D, lane 6 versus 5*). However, DYRK1A overexpression in neurons significantly elevated the ratio of pAKT Ser-473/AKT to 160.2 ± 12.6% of control (Fig. 7, *A* and *C, lane 8 versus lane 7*, *p* = 0.0087). Moreover, DYRK1A elevated to the ratio of pGSK3β Ser-9/GSK3β to 196.2 ± 10.7% of control (Fig. 7, *A* and *D, lane 8 versus 7*, *p* = 0.0004), indicating that DYRK1A ameliorated insulin resistance in neurons.

DYRK1A inhibitor harmine was used to further confirm DYRK1A's promotion of insulin signaling in primary neurons. IRS-1 was increased to 188.4 ± 4.4% of control by acute insulin treatment (Fig. 7, *E* and *F, lane 2 versus 1*, *p* < 0.0001). Harmine treatment decreased the IRS-1 expression and abolished the IRS-1 increase induced by acute insulin treatment (Fig. 7, *E* and *F, lanes 1-4*). At the insulin-resistant state, IRS-1 could not be increased by acute insulin stimulation and harmine treatment further decreased IRS-1 expression (Fig. 7, *E* and *F, lanes 4-8*). Similar with findings in SY5Y, insulin sensitivity was attenuated in the harmine-treated group compared with the control group indicated by the decrease of ratio changes of pAKT Ser-473/AKT (reduced to 55.1 ± 2.8%, *p* = 0.0011) and pGSK3β Ser-9/GSK3β (reduced to 84.6 ± 1.3%, *p* = 0.0046). In the insulin-resistant state, the ratio of pAKT Ser-473/AKT and pGSK3β Ser-9/GSK3β did not change with or without harmine treatment, indicating the unresponsiveness of neurons to insulin stimulation (Fig. 7, *E*, *G,* and *H*).

## Discussion

Here we identified DYRK1A as a novel regulator of the IRS-1 protein. We found that DYRK1A interacted and phosphorylated IRS-1. DYRK1A up-regulated IRS-1 expression through stabilizing its protein. DYRK1A and IRS-1 were coordinately up-regulated in the insulin-resistant PFC of *db*/*db* mice. Moreover, DYRK1A ameliorated insulin resistance in SH-SY5Y and rat primary neurons. Accumulating studies have demonstrated that aberrant IRS-1 expression including total protein down-regulation and abnormal phosphorylation play crucial roles in insulin resistance (44, 48); however, the underlying mechanisms of IRS-1 dysregulation are still unclear. Our study here indicated that DYRK1A plays important roles in regulation of IRS-1 protein and insulin signaling.

As a Ser/Thr protein kinase, DYRK1A exerts most of its function by phosphorylating various downstream substrates. Similarly, we found that DYRK1A could phosphorylate IRS-1. We showed that DYRK1A induced a pronounced elevation of Ser-312 and Ser-616 phosphorylation in IRS-1. S312A/S616A mutants still responded to DYRK1A, indicating that there are other phosphorylation sites unidentified. More investigations are necessary to further elucidate the phosphorylation of IRS-1 by DYRK1A.

Decreased IRS-1 expression has been characterized in insulin resistance. Several studies demonstrated drugs that up-regulate IRS-1 expression exert the function of alleviating insulin resistance (49, 50). Consistent with this, our data demonstrated that DYRK1A ameliorated insulin resistance in both SY5Y and primary neurons by elevating IRS-1 protein. But it should be noted that the increase of AKT pSer-473 and GSK3β pSer-9 by DYRK1A in IR is only moderate, implying that DYRK1A can only partially, but not completely reverse IR. DYRK1A increase could not completely offset the influence of IR, and even worse, increase of DYRK1A could exacerbate the AD pathology by phosphorylating several key molecules in AD such as APP, Tau, and PS1. Future studies are needed to further clarify the underlying mechanisms.

DYRK1A haploinsufficient mice have a low functional β-cell mass (51, 52). Inhibition of DYRK1A in the pancreas was shown to promote pancreatic β-cells proliferation (34–37). The discrepancies in those experimental settings imply that different tissues and developmental stages exhibit different regulations of insulin signaling. Consistent with this, our *in vivo* study showed that insulin signaling may vary between different regions of mice brain. Elevations of pAKT Ser-473 and pGSK3β Ser-9 in PFC of *db*/*db* mice compared with *m*/*m* mice at 12 weeks of age suggested the existence of dysfunctions of insulin signaling. DYRK1A and IRS-1 were coordinately increased in the PFC region of *db*/*db* mice at 12 weeks of age. Due to the compromised development of the nervous system in *db*/*db* mice, it would be advantageous to further explore these phenomena in diet-induced obese mice. Furthermore, it would be interesting to further study the interaction of DYRK1A and IRS-1 in distinct brain regions and how the interaction affect insulin signaling with aging.

## Experimental procedures

### Ethical statements

All procedures involving the use of laboratory animals were approved by the Animal Care and Protection Committee of Shandong University and Institutional Ethics Committees of Qilu Hospital, in accordance with the ARRIVE guidelines.

### Cell culture

HEK293 and human neuroblastoma SH-SY5Y cells were cultured in high glucose DMEM (Thermo, Carlsbad, CA) supplemented with 10% fetal bovine serum (Thermo) and 100 units/ml of penicillin and 0.1 mg/ml of streptomycin. Rat primary neurons were isolated from E18 pregnant rats (Experimental Animal Center of Shandong University) and cultured as previously described (53). All cells were maintained in a 37 °C incubator containing 5% CO_2_.

### Plasmids construction and transfection

The plasmids used are as follows: pCMV6-entry-DYRK1A/DYRK1A-MycFlag (RC213183, Origene Co., Beijing, China) and pEnter-DYRK1A/DYRK1A-FlagHis (pCMV6-entry-DYRK1A and pEnter) were both digested with AsiSI and MluI, the shorter fragment of pCMV6-entry-DYRK1A and the longer fragment of pEnter were ligated by T4 DNA ligase; pIRS-1-myc was kindly provided by professor Yehiel Zick (The Weizmann Institute of Science, Rehovot, Israel); pEnter-IRS-1/IRS-1-FlagHis (CH893343) from Vigene Biosciences, Jinan, China; pHis-ubi was kindly provided by professor Weihong Song (The University of British Columbia, Vancouver, Canada). All transfections were carried out with Lipofectamine 2000 (11668-027; Invitrogen) according to the manufacturer's instructions.

### Adeno-associated virus (AAV) and infection

Recombinant adeno-associated virus type 2/9 (rAAV-2/9) overexpressing DYRK1A and control was purchased from Vigene Biosciences (Jinan, China). Rat primary neurons were infected at DIV (days *in vitro*) 7 with a multiplicity of infection of 5E4. After 8 h of infection, medium were replaced with fresh medium and incubated for another 3 days before further experiments.

### Western blotting and antibodies

For immunoblotting analyses, cells or mice brain tissues were lysed in 0.1% SDS-RIPA lysis buffer (Beyotime Institute of Biotechnology, Haimen, China) supplemented with protease and phosphatase inhibitors (Roche Applied Science). The lysates were resolved by SDS-PAGE and immunoblotting was performed as described previously (30). Primary antibodies used are: anti-IRS-1 mAb (number 3407, Cell Signaling, Danvers, MA), anti-DYRK1A mAb (7D10) (number WH0001859M1, Sigma-Aldrich); anti-pAKT Ser-473 mAb (number 4060, Cell Signaling); anti-AKT mAb (number 60203-2-Ig, Proteintech, Wuhan, China); anti-pGSK3β Ser-9 mAb (number 5558, Cell Signaling); anti-GSK3β mAb (number 12456S, Cell Signaling); anti-myc tag antibody (number ab9106, Abcam, Shanghai, China), anti-FLAG mAb M2 (number F1804, Sigma-Aldrich), anti-β-actin mAb (number A1978, Sigma-Aldrich), anti-ubiquitin mAb (number 3936, Cell Signaling), anti-phosphoserine mAb (number05–1000X, Sigma-Aldrich). Secondary antibodies used are as follows: IRDye® 800CW goat anti-mouse IgG (number 925-32210, Li-Cor, Lincoln, NE); IRDye® 800CW goat anti-rabbit IgG (number 925-32211, Li-Cor); IRDye® 680RD goat anti-mouse IgG (number 925-68070, Li-Cor); IRDye® 680RD goat anti-rabbit IgG (number 925-68071, Li-Cor). Detection was performed with the Li-Cor Odyssey imaging system and quantitated with ImageJ software.

### Cycloheximide (CHX) pulse-chase assay

HEK293 cells were transfected with pEnter-IRS-1 with or without pCMV6-entry-DYRK1A. For measuring the influence of harmine (Aladdin, Shanghai, China) on IRS-1 degradation, HEK293 cells were transfected with pIRS-1-myc and treated with DMSO (number D4540, Sigma-Aldrich) or 1 μm harmine for 24 h before harvest. Twenty-four hours after transfection, cells were treated with 300 μg/ml of CHX (MCE, Shanghai, China) and harvested after 0, 12, and 24 h, respectively. Western blotting was used to detect the protein level of IRS-1, and half-life of IRS-1 was calculated using *t*_½_ = −0.693/*ke*, where *ke* is the regression slope of the IRS-1 level to times.

### IP and co-immunoprecipitation (co-IP)

For IP assay, cells were harvested and lysed with RIPA lysis buffer. Lysates were incubated with primary antibody and protein A/G-agarose beads at 4 °C overnight to precipitate target protein. For co-IP assay, cells were harvested and lysed in 1 ml of 1% Nonidet P-40 lysis buffer (1% Nonidet P-40, 50 mm Tris base, 150 mm NaCl, pH 7.4) supplemented with protease inhibitors (Roche Applied Science). Cell lysates were then incubated with anti-c-myc affinity gel (number A7470, Sigma-Aldrich) or anti-FLAG M2 affinity gel (number A2220, Sigma-Aldrich) at 4 °C overnight. Mouse or rabbit IgG (Beyotime Institute of Biotechnology, Haimen, China) was used with protein A/G-agarose beads (Santa Cruz Biotechnology, Santa Cruz, CA) as negative controls. The next day, the beads were washed and boiled as previously described and precipitated proteins were then analyzed by Western blot with the indicated antibodies (30).

### Immunohistochemistry

Immunofluorescence was performed as previously described (54). Mouse anti-DYRK1A mAb (7D10) (number WH0001859M1, Sigma-Aldrich) and Alexa Fluor 594-conjugated goat anti-mouse IgG (number SA00006-3, proteintech, Wuhan, China) were used to detect DYRK1A. Rabbit anti-IRS-1 mAb (number 3407, Cell Signaling, Danvers, MA) and Alexa Fluor 488-conjugated AffiniPure goat anti-rabbit IgG (number SA00006-2, Proteintech, Wuhan, China) were used to detect IRS-1. DAPI (number D9542; Sigma-Aldrich) was used to detect the nucleus. Images were captured using a LSM 780 fluorescent microscope (Carl Zeiss, Jena, Germany) and analyzed with ZEN software.

### In situ PLA

The PLA was performed with a DuoLink® PLA kit (number DUO92101, Sigma-Aldrich), and following the manufacturers' protocol. In brief, after blocking and incubating with mouse anti-DYRK1A and rabbit anti–IRS-1 primary antibodies, the slips were washed and then incubated with PLA probes. The probes were then ligated by the ligation reagent. Next, the amplification reagent containing polymerase and oligonucleotides labeled with detectable fluorophores was applied. After wash, cells were incubated with Alexa Fluor® 488 conjugate α-tubulin (number 5063, Cell Signaling). Finally, the slips were washed and mounted with Duolink® *in situ* mounting medium containing DAPI. For negative controls, IRS-1 antibody was substituted with anti-rabbit IgG antibody, or DYRK1A antibody substituted with anti-mouse IgG antibody. Z-stack images were captured using LSM 780 fluorescent microscope (Carl Zeiss, Jena, Germany) and the maximum intensity projections were obtained by ZEN software.

### Protein expressions of DYRK1A and IRS-1 in the brain of diabetes mouse model

The *db*/*db* mouse model of leptin deficiency is currently the most widely used mouse model of T2DM. Twelve-week-old male *Lepr db*/*db* (*db*/*db*) and *Lepr m*/*m* (*m*/*m*) mice in a C57BLKS/J background were acquired from the Model Animal Research Center of Nanjing University. Body weight was recorded and fasting blood glucose was measured by ACCU-CHEK Active Blood Glucose Meter (Roche Applied Science). Mice were euthanized with an overdose of pentobarbital sodium, hippocampus and PFC were carefully dissected from the brain. Those tissues were lysed in 0.1% SDS-RIPA lysis buffer and TRIzol reagent (Invitrogen) for protein and mRNA expression detection, respectively.

### Total RNA extraction and Real-time RT-PCR

Total RNA was isolated from mice brain tissues by TRIzol reagent and cDNA was synthesized from an equal amount of total RNA utilizing reverse transcription kit (Takara, Japan) as per the manufacturer's instructions. Real-time RT-PCR was performed on ABI 7900HT Fast Real-Time PCR System (Foster City, CA) with SYBR Green Real-time PCR Master Mix (Toyobo, Japan). A comparative CT method (2^−ΔΔ^*^CT^*) was used to analyze IRS-1 and DYRK1A expression levels, β-actin was used as the internal control. Primers used are as follows: IRS-1 (103 bp), forward, 5′-TCTACACCCGAGACGAACACT-3′, and reverse, 5′-TGGGCCTTTGCCCGATTATG-3′; DYRK1A (114 bp), forward, 5′-GCCAGCCGAGCATAAGTGA-3′, and reverse, 5′-GCATCCGCCTCTGTAACATGA′; β-actin (141 bp), forward, 5′-GACAGGATGCAGAAGGAGATTACT-3′; and reverse, 5′-TGATCCACATCTGCTGGAAGGT-3′.

### Establishment of IR cell models and measurement of insulin sensitivity

Insulin resistance cell models were achieved by chronic high insulin pretreatment. For SH-SY5Y cells, culture media were changed to serum-free DMEM containing 1 μm insulin for 24 h. For primary neurons, to exclude the effect of insulin contained in B27, culture media were changed to treatment media (culture media without B27) containing 3 μm insulin for 24 h. After pretreatment, cells were washed three times with PBS and refreshed in fresh media (treatment media without insulin) for 30 min and then stimulated or not with 10 nm insulin for 15 min. Cells were then harvested and protein levels of pAKT Ser-473, AKT, pGSK3β Ser-9, GSK3β were determined by Western blot. Ratios of pAKT Ser-473/AKT and pGSK3β Ser-9/GSK3β were calculated and the ratio changes after acute insulin re-stimulation were used to reflect cell responsiveness to insulin.

### Statistics

Data are presented as mean ± S.D. from three independent experiments. Differences between two groups were assessed by Student's *t* test, and those among more than two groups were performed with ANOVA followed by Tukey's multiple comparisons test. Data from animals are presented as mean ± S.D. Correlations between IRS-1 and DYRK1A proteins were determined by Pearson's correlation test. *p* < 0.05 was considered to be significant. All analyses were performed with Prism (GraphPad Software, Inc., San Diego, CA).

## Author contributions

S. T. and X. S. data curation; S. T. and X. S. software; S. T. and X. S. formal analysis; S. T. and X. S. investigation; S. T. and X. S. visualization; S. T. and X. S. methodology; S. T. and X. S. writing-original draft; S. T. project administration; S. T. and X. S. writing-review and editing; W. J., M. L., J. Z., and X. S. resources; M. L. and X. S. conceptualization; X. S. supervision; X. S. funding acquisition; X. S. validation.

## Footnotes

2 The abbreviations used are:

AD: Alzheimer's disease
T2DM: type 2 diabetes mellitus
IR: insulin receptor
IRS: insulin receptor substrate
DYRK1A: dual-specificity tyrosine phosphorylation-regulated kinase 1A
PFC: prefrontal cortex
PLA: proximal ligation assay
IP: immunoprecipitation
DMEM: Dulbecco's modified Eagle's medium
AAV: adeno-associated virus
CHX: cycloheximide
mAb: monoclonal antibody
GSK3β: glycogen synthase kinase 3β
DAPI: 4′,6-diamidino-2-phenylindole
ANOVA: analysis of variance.
